# Physiological responses to low-force work and psychosocial stress in women with chronic trapezius myalgia

**DOI:** 10.1186/1471-2474-10-63

**Published:** 2009-06-07

**Authors:** Anna Sjörs, Britt Larsson, Joakim Dahlman, Torbjörn Falkmer, Björn Gerdle

**Affiliations:** 1 Rehabilitation Medicine, Faculty of Health Sciences, Linköping University, SE 581 85 Linköping, Sweden; 2 Pain and Rehabilitation Centre, University Hospital, SE 581 85 Linköping, Sweden; 3 Department of Rehabilitation, School of Health Sciences, Jönköping University, Box 1026, SE 551 11 Jönköping, Sweden; 4 School of Occupational Therapy and Social Work, Curtin University of Technology, GPO Box U1987, Perth, WA 6845, Australia

## Abstract

**Background:**

Repetitive and stressful work tasks have been linked to the development of pain in the trapezius muscle, although the underlying mechanisms still remain unclear. In earlier studies, it has been hypothesized that chronic muscle pain conditions are associated with imbalance in the autonomic nervous system, predominantly expressed as an increased sympathetic activity. This study investigates whether women with chronic trapezius myalgia show higher muscle activity and increased sympathetic tone at baseline and during repetitive low-force work and psychosocial stress, compared with pain-free controls.

**Methods:**

Eighteen women with chronic trapezius myalgia (MYA) and 30 healthy female controls (CON) were studied during baseline rest, 100 min of repetitive low-force work, 20 min of psychosocial stress (Trier Social Stress Test, TSST), and 80 min recovery. The subjects rated their pain intensity, stress and energy level every 20 min throughout the experiment. Muscle activity was measured by surface electromyography in the trapezius muscle (EMGtrap) and deltoid muscle (EMGdelt). Autonomic reactivity was measured through heart rate (HR), skin conductance (SCL), blood pressure (MAP) and respiration rate (Resp).

**Results:**

At baseline, EMGtrap, stress ratings, and HR were higher in MYA than in CON. Energy ratings, EMGdelt, SCL, MAP and Resp were, however, similar in the two groups. Significant main group effects were found for pain intensity, stress ratings and EMGtrap. Deltoid muscle activity and autonomic responses were almost identical in MYA and CON during work, stress and recovery. In MYA only, pain intensity and stress ratings increased towards the end of the repetitive work.

**Conclusion:**

We found increased muscle activity during uninstructed rest in the painful muscle of a group of women with trapezius myalgia. The present study could not confirm the hypothesis that chronic trapezius myalgia is associated with increased sympathetic activity. The suggestion of autonomic imbalance in patients with chronic local or regional musculoskeletal pain needs to be further investigated.

## Background

Chronic myalgia is a complex and multifactorial condition, affecting significantly more females than males, whose etiology and pathophysiology are sparsely known. Musculoskeletal pain is often exacerbated by mental and social stress and it is suggested that psychophysiological mechanisms play an important role in the development and maintenance of chronic pain states [1]. Passatore and Roatta [2] advocate that stress may facilitate the development of chronic pain states, irrespective of their origin.

There is a growing body of evidence for high quantitative demands, lack of support from colleagues, low job control and low influence being related to the development of neck pain [3]. Evidence for a relation between mental stress at work and upper extremity complaints has been reported by Malchaire et al. [4] and Bongers et al. [5]. Mental stressors are thought to increase the risk of developing a musculoskeletal disorder in the neck/shoulder region, particularly so in occupations of low physical demand [6].

It has been proposed that low load repetitive work promotes over-activity of low threshold motor units resulting in muscle morphological changes, fatigue and pain [6]. Surface electromyography (EMG) can be used to investigate force and endurance (fatigue) aspects of muscles. Altered neuromuscular control in patients with pain has been a focus both in research and in clinical practice during several years. Using EMG, certain aspects of the neuromuscular control such as muscle relaxation and synchronization of activity between muscles have been investigated [7]. For example, based on clinical observations that patients with myalgia have tender muscles it is often assumed that a vicious circle of pain and hyperactivity exist in chronic pain. The supposed increased muscle tension is clinically targeted for intervention with the purpose of reducing pain. However, research using EMG shows a more complex situation.

Acute nociception/pain can lead to altered sharing *between *muscles within an anatomical region, but also to a changed spatial distribution of EMG activity *within *a muscle, i.e., trapezius [8,9]. During dynamic muscle contractions, increased EMG activity has generally been found in parts of the contraction cycle [10-16]. Several of these studies relate their results to the *pain-adaptation model *[17]. According to this model, a decrease in agonist muscle activity and an increase in antagonist muscle activity will be found as a consequence of nociception. There are, however, indications that during maximal contractions local pain inhibits activity specifically of painful muscles but not activity of pain free synergistic muscles [18].

Previous studies have investigated the influence of mental stress on EMG activity of the trapezius muscle, and a significant increase in trapezius EMG activity has been found during mental stress and cognitive task performance [6,19]. Moreover, Lundberg et al. [20] found higher levels of trapezius muscle activity during mental and physical work stress in people with trapezius myalgia and substantial neck/shoulder pain than in pain-free individuals.

Several models of the pathophysiology of chronic pain pay attention to the autonomic involvement in the pathogenesis [2,21]. Altered activity in the sympathetic nervous system, i.e., increased or decreased reactivity in response to stimuli, has been implicated in the genesis of muscle pain [22]. Sympathetic involvement in the activation of muscle fibers is a potential explanation for the association between altered autonomic activity and the development of musculoskeletal pain. A recent study [23] has shown that the sympathetic nervous system modulates the contractility of skeletal muscle fibers, providing evidence for a link between the autonomic and motor systems. Furthermore, the autonomic nervous system is believed to undergo plastic changes in chronic pain states [24]. There is evidence that the autonomic state of patients with fibromyalgia, i.e., persistent generalized pain and hyperalgesia, is characterized by increased sympathetic and decreased parasympathetic tone at baseline [25], with concurrent sympathetic hyporeactivity to various stressors [26]. However, little is known about the autonomic regulation in patients with local or regional pain. Previous studies of whiplash associated disorders and chronic low back pain have shown indications of increased sympathetic and decreased parasympathetic activity, which could be a sign of autonomic imbalance [22,27].

Although several studies have been published on the topic, mainly focusing on widespread pain, the mechanisms behind initiation and maintenance of chronic musculoskeletal pain still remain unclear. The potential link between muscle over-activity and development of pain in the neck/shoulder region is yet to be confirmed. Earlier laboratory studies have used functional tests, low-grade mental stress or repetitive tasks of short duration to investigate if muscle activity or sympathetic activity is altered in patients with chronic musculoskeletal pain. Contradictory results have been reported and in order to explore potential alterations/dysfunctions further, we used repetitive work tasks of longer duration and a powerful psychosocial stressor in this study.

The aim of this study was to assess whether women with chronic trapezius myalgia show different physiological reactions, compared with pain-free controls, during experimental repetitive low-force work and a standardized psychosocial stress test. Although the study was mainly explorative, it was hypothesized that the chronic pain patients would show higher trapezius muscle activity and increased sympathetic tone at baseline and in response to repetitive work tasks and psychosocial stress.

## Methods

### Subjects

#### Subjects with trapezius myalgia

In order to recruit subjects with trapezius myalgia (denoted MYA), the medical reports of former female outward patients who had been referred to the multidisciplinary Pain and Rehabilitation Centre at Linköping University Hospital due to: neck myalgia and with the international classification of diseases (ICD) number M 79.1, or cervicalgia ICD number M 54.2, or cervico-brachial syndrome ICD number M 53.1 and with no other diagnosis were identified. Invitation letters with information about the study were sent to 220 former patients. Those who volunteered to participate were contacted by telephone and 24 of them were invited to be examined by a standardized clinical neck and shoulder examination [28] and to complete the Nordic Ministry Council Questionnaire (NMCQ), which was used to survey their present pain [29]. The clinical examination includes questions on pain, tiredness and stiffness on the day of examination, as well as physical tests including; range of motion and tightness of muscles, pain threshold and sensitivity, muscle strength and palpation of tender points. Diagnosis of trapezius myalgia includes: neck pain at the examination day, tightness of the trapezius muscle, i.e., a feeling of stiffness in the descending region of the trapezius muscle reported by the subject at examination of lateral flexion of the head, and palpable tender parts in the trapezius muscle. Range of motion of the cervical columna is to be normal or slightly decreased. The examiner was a physician (BLa), specialized in occupational medicine. The examiner was aware of to which group the participants belonged.

Eligible subjects were those women who reported pain in the descending region of the trapezius muscle during the last seven days and reported neck and shoulder pain more than 90 days over the last 12 months. Moreover, subjects should not report pain during the last seven days from more than three body regions according to the NMCQ.

The following exclusion criteria were used: 1) signs of tendinitis or joint affections in the shoulders, 2) prior neck trauma, 3) rheumatoid arthritis or other systemic diseases, 4) neurological diseases, 5) metabolic diseases, 6) fibromyalgia syndrome (determined by tender point examination and pain drawing according to the ACR criteria of 1990) [30]. The exclusion criteria were assessed by interview at the first telephone contact and by examination.

Through these criteria eighteen women with chronic trapezius myalgia (MYA) were recruited for the study (Table 1). Two subjects reported that their pain was intermittent and 16 reported constant pain.

**Table 1:** Characteristics for 18 subjects with chronic trapezius myalgia (MYA) and 30 pain-free controls (CON).

	**MYA**	**CON**	
	Mean (SD)	Mean (SD)	p-value
Age	40.0 (6.0)	39.9 (5.6)	>.3
Height (cm)	168.4 (4.7)	167.5 (5.0)	>.3
Weight (kg)	74.8 (11.2)	67.0 (9.4)	.014
BMI (kg/m^2^)	26.4 (3.7)	23.8 (2.9)	.012
			
**MYA group's pain history and pain intensity**			
	Median	Range	
Months with pain	126	37 – 273	
Months with chronic pain	101	36 – 273	
VAS neck	70	40 – 90	
VAS shoulders	67	19 – 88	
VAS arms	59	0 – 72	
VAS hands/wrists	49	0 – 86	
VAS upper back	42	0 – 77	
VAS lower back	26	0 – 85	
VAS hips	3	0 – 84	
VAS knees	0	0 – 78	
VAS feet	0	0 – 78	

Pain intensity was measured using visual analogue scale (VAS) and concerned the previous 30 days.

#### Healthy controls

Thirty age-matched healthy women with no neck/shoulder pain (denoted CON) comprised the control group (Table 1). The control subjects were recruited via advertisements in daily newspapers. They were investigated using brief versions of the clinical examination. Exclusion criterion, in addition to the above mentioned, was the presence of pain in the neck/shoulder region for more than 2–3 days during the last 12 months.

#### Ethics

After receiving verbal and written information about the study, all MYA and CON subjects signed a consent form that was in accordance with the Declaration of Helsinki. The study was granted ethical clearance by the Linköping University Ethics Committee (Dnr M46-07).

### Procedure

In the experimental session, the subjects reported to the laboratory in the morning. The subjects were asked not to use any medications two days before the experimental day, except paracetamol preparations if needed, and to refrain from intake of caffeine and nicotine 12 hours prior to the study. Subjects were also instructed not to perform any strenuous exercise of the neck/shoulders on the day preceding the experiment. Firstly, one of the physicians (BGe or BLa) met the subject during approximately 15 min. It was checked that she had understood the experimental part of the study and still wanted to participate, that no important changes had occurred in her medical status and that the instructions with respect to pharmacological treatment, exercise, food intake and caffeine/nicotine had been followed.

Thereafter, two custom-made microdialysis catheters were inserted into the trapezius muscle at a standardized anatomical point on the most painful side for the MYA group and on the dominant side for the CON group, as described by Larsson et al. [31]. Results from microdialysis and details regarding the microdialysis method will be presented elsewhere. The experiment started with a 120-min resting period to allow the tissue to recover from possible changes in the interstitial environment caused by the minimal trauma of catheter insertion [32]. Equipments for blood pressure measurements and continuous physiological recordings were fitted to the subject. During this resting phase, the subject was given a standardized meal, which was served 80 min after start of the experiment.

**Figure 1:**
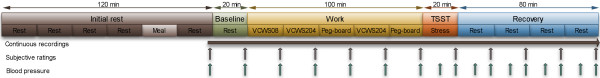
**Experimental protocol**. The continuous recordings were electromyogram, electrocardiogram, skin conductance and respiration. Subjective ratings comprised pain intensity ratings and the stress-energy questionnaire.

The data acquisition began with a 20-min baseline period – still at rest. During baseline, the subjects were seated in a comfortable chair and were told to avoid movements activating the neck/shoulder muscles. A low-force repetitive work was then performed for 1 h and 40 min (Figure 1), utilizing three work stations described below. The purpose was to exacerbate pain in the trapezius myalgia group, especially in their most painful trapezius muscle, by performing repetitive exercises predominantly with their most painful side.

During the work period, the three work stations were alternated in 20-min intervals starting with Simulated Assembly, that was performed only once, followed by the Fine Finger Dexterity and peg-board exercises that both were performed twice. Following these exercises, the subjects performed the Trier Social Stress Test (TSST). The experiment ended with an 80-min recovery period at rest.

Pain was rated shortly after catheter insertion and then every 20 min throughout the experiment, starting at the baseline period. Electrocardiogram, skin conductance, respiration, and surface electromyogram signals were continuously recorded, starting at the baseline period. Blood pressure measurements were made every 20 min during baseline and work periods, increasing to every 10 min during TSST and recovery.

### Work stations

Two standardized work stations (Valpar Component Work Stations, VCWS; Valpar, Tucson, USA) and one peg-board exercise, previously described by Rosendal et al. [32] were utilized. The work pace for each station was determined by pilot trials and set to meet the requirements in the VCWS protocols.

The VCWS08, Simulated Assembly, is a repetitive assembly work requiring manipulation and bilateral use of the upper extremities. The work sample exercise is characteristic for conveyor belt-assembly jobs, in which the product moves towards and away from the workers on an assembly line. The subject was positioned in front of the work sample and, as the assembly wheel turned at a constant speed, she made as many three-part assembly operations as possible within the 20 min time limit. First, the subject placed a pin in a hole on the assembly wheel. Next, she placed a spacer on the pin and finally a cap on top of the spacer. Correct assemblies were automatically counted and recycled back into part bins at the front of the work sample. The subjects were instructed to keep a rate of 13 assemblies per minute, which was checked by the experimenter.

The VCWS204, Fine Finger Dexterity work sample, simulates sedentary work and, in this study, two exercises were performed. In the first exercise, the subject used her hand on the dominant/most painful side to turn five grooved metal rods (finger screws) into a bar and then turn the five screws all the way out of the bar again, as quickly as she could. In the second exercise, wiring, the subject threaded a nylon wire through 20 metal pins that first needed to be lifted out of holes and held in place by reverse-tension tweezers as the wire was threaded. The subjects were instructed to work as fast as they could and to strive for two or more complete cycles during the work period.

The peg-board exercise, a repetitive arm movement task that was performed with the dominant/most painful arm, consisted of moving short wooden sticks (11.8 g) back and forth between standardized positions 30 cm apart on a pegboard at a frequency of 1 Hz indicated by an electronic metronome (Korg Inc., Tokyo, Japan); for details see [32].

### TSST

The Trier Social Stress Test (TSST) is a valid and reliable standardized psychosocial stressor, which was first described by Kirschbaum et al. [33]. The TSST protocol consists of a 10-min preparatory and information period, a 5-min speech and a 5-min verbal arithmetic task. For all subjects, the TSST took place between 1.30 pm and 3.30 pm to minimize confounds from diurnal variation in hormone levels. The experimental sessions were scheduled day 1–10 in the menstrual cycle, i.e., the follicular phase, since cortisol reactivity to the TSST changes over the menstrual cycle [34].

Summarized, the subject was led to the TSST room where she was instructed to stand behind a microphone in front of a committee, consisting of two men and one woman. The experimenter instructed the subject to deliver a 5-min speech as for a job application, for which she had approximately 5 min to prepare, and that a second task would follow. After the job interview, the subject had to solve a verbal arithmetic task, in which she had to count backwards from 1687 in steps of 13 as quickly and correctly as possible. In case of miscalculation, the subject had to start over from 1687. The verbal arithmetic task also lasted 5 min. Before starting, the subject was informed that the whole session would be videotaped and voice recorded, and that the committee was trained in behavioral observation. This incorrect information was given as an additional stressor and was explained to the subject in a debriefing session after completion of the TSST.

### Measurements

#### Pain ratings

Throughout the experiment, the subjects were asked to rate their pain intensity on a graphic rating scale, i.e., a visual analogue scale with numbers (0 – 10) provided along the scale for guidance. The scale was drawn on a 100-mm line and anchored with "no pain" and "worst possible pain". All pain ratings concerned pain in the trapezius muscle of both the dominant (for CON) or most painful (for MYA) side (PAINdomp) and the contralateral side (PAINclat). Pain ratings were obtained at the end of each 20-min period.

#### Stress-Energy questionnaire

Every 20 min throughout the experiment, subjects completed the Stress-Energy Questionnaire[35], which is an instrument with two scales that measure two critical aspects of mood at work. The Stress scale represents a dimension ranging from positively evaluated low activation (relaxed) to negatively evaluated high activation (distressed), whereas the Energy scale deals with the dimension ranging from negatively evaluated low activation (dull) to positively evaluated high activation (enthusiastic). The instrument includes 12 adjectives, six in each dimension. Three adjectives within each dimension are positively loaded and three are negatively loaded. The checklist uses a six-point response scale (0–5) for each item, ranging from "not at all" to "extremely". The following items are included: "rested", "relaxed" and "calm" (low stress); "tense", "stressed" and "pressured" (high stress); "active", "energetic" and "focused" (high energy); "dull", "inefficient" and "passive" (low energy).

Stress and energy scores are calculated as mean ratings of the six items after reversal of the items standing for low stress and low energy. High values thus indicate a high stress and high energy level, respectively. For the Stress scale, the neutral point (neither stressed nor calm) has been calculated to be 2.4, and for the Energy scale the corresponding value is 2.7. The Stress-Energy questionnaire is a valid instrument for assessing stress at work [36].

#### Electromyogram

Surface electromyogram (EMG) signals were recorded from the descendent part of the trapezius muscle and the deltoid muscle on the dominant/most painful side using surface electrodes positioned according to SENIAM recommendations [37]. The skin was first dry shaved and then cleaned with an alcohol and ether solution (4:1). Two recording silver-chloride electrodes (Ambu, Ballerup, Denmark), with a diameter of 7 mm, abraded with redux paste, were placed 20 mm apart (center to center distance)on the skin. A reference electrode was attached over the process spinosus at C7 level.

The electric signals were recorded with a digital wireless acquisition system featuring differential high impedance (>10 GΩ) inputs, -50 mV to +35 mV range, 0–280 Hz bandwidth, and <3 μV_RMS _noise. Each channel was A/D converted with 16 bit resolution at 1,000 samples per second, and the data were stored on a computer. The EMG recording system was custom made by the Department of Biomedical Engineering and Informatics, University Hospital, Umeå, Sweden. When the electrodes were applied, the signal quality was checked visually on the screen of the PC. The recording of EMG data started at the baseline period and continued throughout the experiment. For the recordings during the experiment, 5-min segments of EMG data were collected from the middle of each 20-min period.

Before the experiment, reference recordings were performed to relate the EMG activity during the experiment, since individual differences in muscle size, thickness of skin and subcutaneous fat etc. can influence the signal strength. The reference recordings also provide a possibility to investigate the subject's ability to relax the trapezius muscle when instructed to do so. The subject was first instructed to relax the neck/shoulder muscles completely with her hands passively resting on the thighs while sitting comfortably. Thereafter, the subject performed two brief (5–6 sec) static shoulder forward flexions (90 degrees) with a 2 kg dumbbell, resting 1–2 minutes between the contractions. The reference recordings ended with another neck/shoulder relaxation. From these recordings, four 5-sec segments were selected for analysis, i.e., two relaxation segments and two contraction segments.

EMG data were high pass filtered with 6:th order digital Butterworth filters cutting off frequencies below 15 Hz. In order to suppress distinct interference from the power mains, 1 Hz wide 3:rd order Butterworth notch filters centred at 50 and 100 Hz respectively were applied. Root mean square (RMS) amplitude (μV) was calculated in the time domain for each 5-min segment from the experiment and 5-sec segment from the reference recordings. The variables were denoted EMGtrap for the trapezius muscle and EMGdelt for the deltoid muscle. Prior to filtering and RMS calculation, the raw data were checked for amplifier output clipping due to large DC levels appearing if the subjects happened to squeeze the EMG electrodes: data points being closer than 0.5 mV to maximal positive or negative amplifier input range, and their neighbour data points, 1 s before and 1 s after, were considered unreliable and were discarded. In total, 5.5% of the trapezius EMG data and 5.8% of the deltoid EMG data were discarded.

#### Electrocardiogram, skin conductance and respiration

Electrocardiogram (ECG), skin conductance level (SCL) and respiration measurements were made using ProComp Infiniti recording system (Thought Technology, Montreal, Canada), an 8-channel recording system with 14 bit resolution. After filtering and amplification, data were digitized and transmitted via Bluetooth for real-time presentation and storage on a computer. SCL and respiration measures were sampled at 32 Hz, whereas ECG was sampled at 256 Hz.

The ECG was recorded with a standard lead II placement, using three electrodes placed on the left and right clavicles and on the lower left side of the chest. The electrodes used were disposable pre-gelled Ag/AgCl electrodes. Channel input range was 0–12 mV_RMS _with a bandwidth of 0.05 Hz-1 kHz and accuracy ± 3 μV_RMS_. The negative electrode was placed on the right clavicle, the ground electrode on the left clavicle and the positive electrode was placed on the lower left side of the chest. Heart rate (HR, beats/min) was calculated via R-wave detection.

SCL was measured with two disposable pre-gelled Ag/AgCl electrodes (1 cm contact area diameter) on the thenar and hypothenar eminences on the non-dominant/least painful side. Channel range was 0–30.0 μS with ± 0.2 μS accuracy. During SCL recordings, the voltage across the electrodes was held constant at 0.5 V.

Respiration, measured as chest expansion, was recorded using a strain sensitive sensor strapped around the chest. Respiration rate (Resp, breaths/min) was computed breath-to-breath from the respiration signal.

During the experiment, ECG, SCL and respiration data were collected in 5-min segments from each 20-min period and HR and Resp were calculated from each segment. Default 5-min segment was minute 10–14 within the 20-min period, which was chosen in order to minimize disturbances from transportation and other measurements made in the beginning and at the end of each 20-min period.

#### Blood pressure

Blood pressure measurements were carried out with the oscillometric method using an automatic ambulatory blood pressure monitor (90217 ABP monitor; SpaceLabs, Redmond, USA). Measurements were done in the non-dominant/least painful arm and appropriately sized cuffs according to arm circumference were used. Systolic, diastolic and mean arterial blood pressures (MAP, mmHg) were recorded in each measurement. Blood pressure measurements were made every 20 min during baseline and work periods and every 10 min from TSST and onwards.

### Statistical analyses

All statistical analyses were conducted in SPSS version 15.0 for Windows (SPSS Inc.). Kolmogorov-Smirnov tests were performed to identify variables not normally distributed.

As a simple approach to compare the two groups (MYA and CON), independent samples t-tests were performed on baseline data, as well as on mean values for the entire experiment. To assess possible differences between MYA and CON in their autonomic responses and muscle activity responses to psychosocial stress, the measurements taken during TSST were tested. For EMG data, the reference recordings were also compared between groups. Pain ratings and EMG data were not normally distributed according to the Kolmogorov-Smirnov test. Therefore, tests for differences between groups were instead conducted with the Mann-Whitney U-test.

A linear mixed regression model was then fitted using restricted maximum likelihood with pain intensity, stress, energy, EMG, HR, Resp, MAP and SCL as the dependent variable, respectively. In the mixed model analyses, subject (1 to 48) was applied as a random factor and the fixed categorical independent variables were group (MYA and CON) and time (20-min sampling period 1 to 11). Possible interaction between group and time was also assessed and included in the model if the interaction effect was significant. Bonferroni corrected post hoc tests were performed when significant main effects were found. The time dependence within in each subject was modeled as autoregressive with a single time lag (AR [1]). Similar results were obtained when we instead modeled the time dependence as autoregressive moving average with single time lags (ARMA [1,1]); these results are therefore not presented. Weight was applied as a covariate in the mixed model analyses to control for the difference in mean weight between the MYA and CON groups (Table 1). For the analyses of EMG data, all four reference recordings for EMGtrap and EMGdelt respectively were applied as additional covariates. Since EMG data were not normally distributed, EMGtrap and EMGdelt were ln-transformed before analysis.

Correlations between pain intensity, stress ratings and all physiological variables were calculated for each 20 min period using Spearman rank correlations. In all statistical analyses, the two-tailed significance level was set at α = 0.05.

## Results

### Baseline and reference recordings

The MYA group reported significantly higher baseline pain ratings than the controls (Table 2) both in the dominant/most painful side (PAINdomp) and the contralateral side (PAINclat). They also reported higher stress ratings during baseline than the controls but energy ratings did not differ between the groups. Baseline trapezius muscle activity (EMGtrap) was significantly higher in MYA than in CON. For the deltoid muscle, the baseline level of EMGdelt was not significantly different between the two groups. The MYA group had significantly higher HR at baseline compared with the CON group (Table 2). There were no significant baseline differences in SCL, Resp and MAP between the two groups.

**Table 2:** Tests for differences between the chronic pain group (MYA) and controls (CON) in their subjective ratings, muscle activity and autonomic variables.

	**Baseline**			**Overall mean**			**TSST**		
**Measure**	**CON** Mean (SD)	**MYA** Mean (SD)	**p-value**	**CON** Mean (SD)	**MYA** Mean (SD)	**p-value**	**CON** Mean (SD)	**MYA** Mean (SD)	**p-value**
**PAINdomp** (mm)	4 (10)^a^	50 (39)^a^	**<.001**^b^	7 (10)^a^	68 (38)^a^	**<.001**^b^	3 (10)^a^	72 (45)^a^	**.001**^b^
**PAINclat** (mm)	0 (0)^a^	20 (25)^a^	**<.001**^b^	0 (0)^a^	30 (36)^a^	**<.001**^b^	0 (0)^a^	30(49)^a^	**<.001**^b^
**Stress** (S-E score)	.7 (.5)	1.5 (.8)	**<.001**	1.5 (.5)	2.2 (.5)	**<.001**	3.6 (1)	3.7 (.9)	>.3
**Energy** (S-E score)	1.9 (.9)	1.9 (.6)	>.3	2.6 (.5)	2.5 (.4)	>.3	3.4 (.6)	2.9 (.8)	**.015**
**EMGtrap** (μV)	10.4 (17)^a^	25.9 (35)^a^	**.013**^b^	37.6 (42)^a^	56.3 (58)^a^	.055^b^	17.1(41)^a^	23.2 (28)^a^	>.3^b^
**EMGdelt** (μV)	8.8 (15)^a^	11.8 (27)^a^	.103^b^	32.5 (22)^a^	44.7 (42)^a^	>.3^b^	10.1 (9)^a^	10.8 (31)^a^	>.3^b^
**HR** (beats/min)	66.3 (8.3)	72.7 (8.0)	**.013**	77.9 (8.1)	80.5 (10.9)	>.3	94.6 (16)	93.2 (23)	>.3
**SCL** (μS)	5.0 (4.2)	5.9 (5.6)	>.3	10.5 (4.5)	11.4 (2.9)	>.3	14.1 (5)	16.6 (5)	.123
**Resp** (breaths/min)	16.3 (2.2)	16.7 (1.8)	>.3	17.5 (1.9)	17.5 (1.5)	>.3	15.6 (2)	15.1 (2)	>.3
**MAP** (mmHg)	88.3 (7.7)	86.8 (9.4)	>.3	93.5 (6.7)	93.3 (10.4)	>.3	108.6 (12)	106.9 (16)	>.3

Note: ^a^Median (interquartile distance); ^b^Mann-Whitney U-test; domp = dominant/most painful trapezius; clat = contralateral trapezius; EMGtrap = trapezius muscle activity; EMGdelt = deltoid muscle activity; HR = heart rate; SCL = skin conductance level; Resp = respiration rate; MAP = mean arterial pressure.

**Figure 2:**
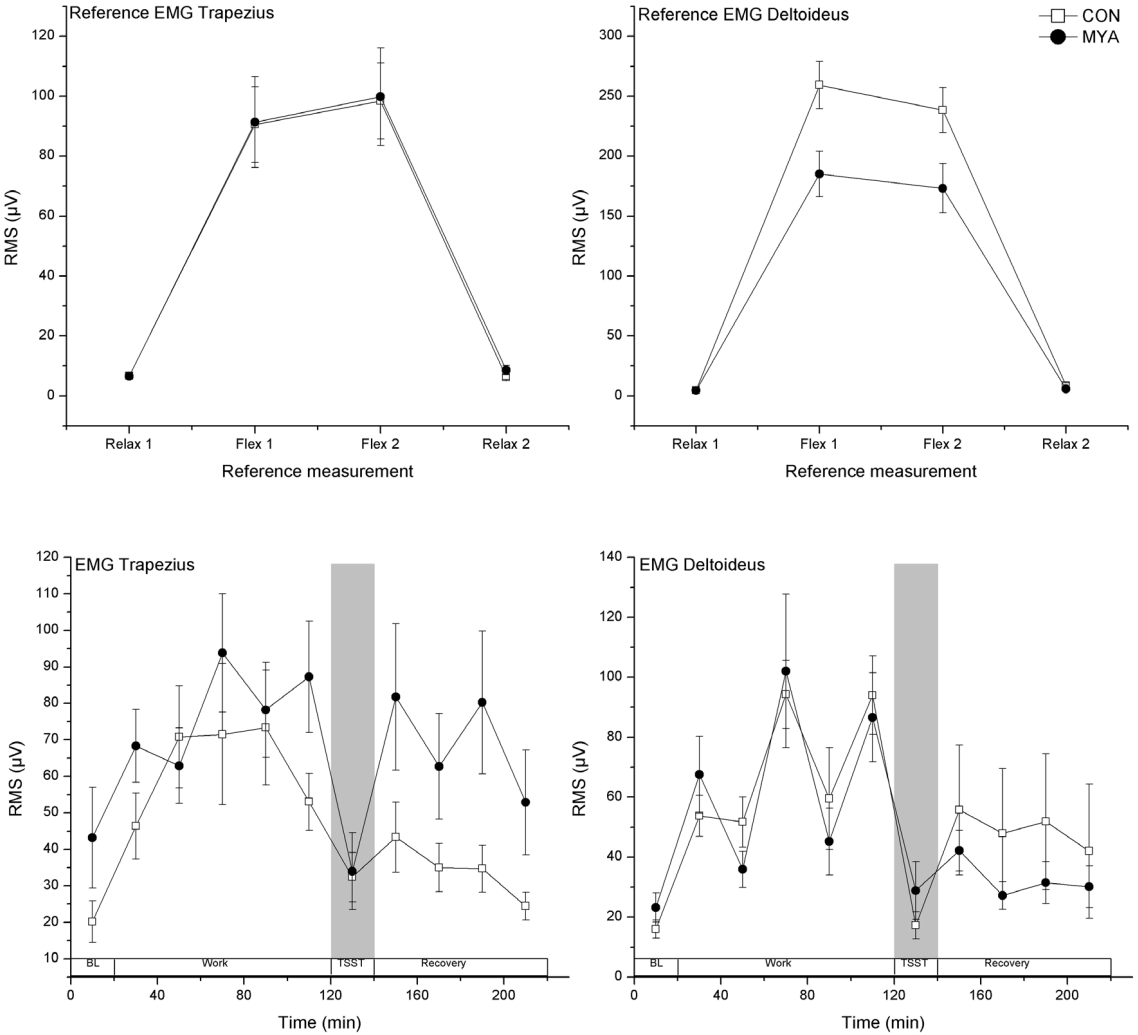
**EMG measured as mean RMS (± SEM) for the reference recordings and for each 20-min period throughout the experiment**. The reference contraction (Flex) was a 90 degrees forward flexion of the arm holding a 2 kg dumbbell. BL denotes baseline.

There were no significant differences between MYA and CON groups in the reference measurements of EMGtrap (Figure 2, p-values from .15 to .95). Likewise, the reference relaxation measurements of EMGdelt were not significantly different between MYA and CON groups (p-values .28 and .47). However, the reference contraction measurements showed that MYA had significantly lower EMGdelt than CON (p-values .009 and .013) when holding a dumbbell at 90 degrees forward flexion.

### Overall mean

Mean pain ratings over the entire experiment were significantly higher in the MYA-group compared with the CON-group for both PAINdomp and PAINclat (Table 2). Stress ratings were also higher for the trapezius myalgia group than the controls throughout the experiment. The differences in mean EMGtrap from the entire experiment did not reach statistical significance. Likewise, the overall means for Energy, EMGdelt, HR, SCL, Resp and MAP were not significantly different between the two groups.

### TSST

Tests for differences between the two groups during TSST revealed significantly higher pain ratings and significantly lower Energy ratings in MYA (Table 2). Stress ratings and physiological measurements did not differ between the two groups during the psychosocial stress test.

### Mixed model analyses

#### Pain ratings

Pain ratings for both sides were, as expected, significantly higher in MYA than CON at all time points.

**Figure 3:**
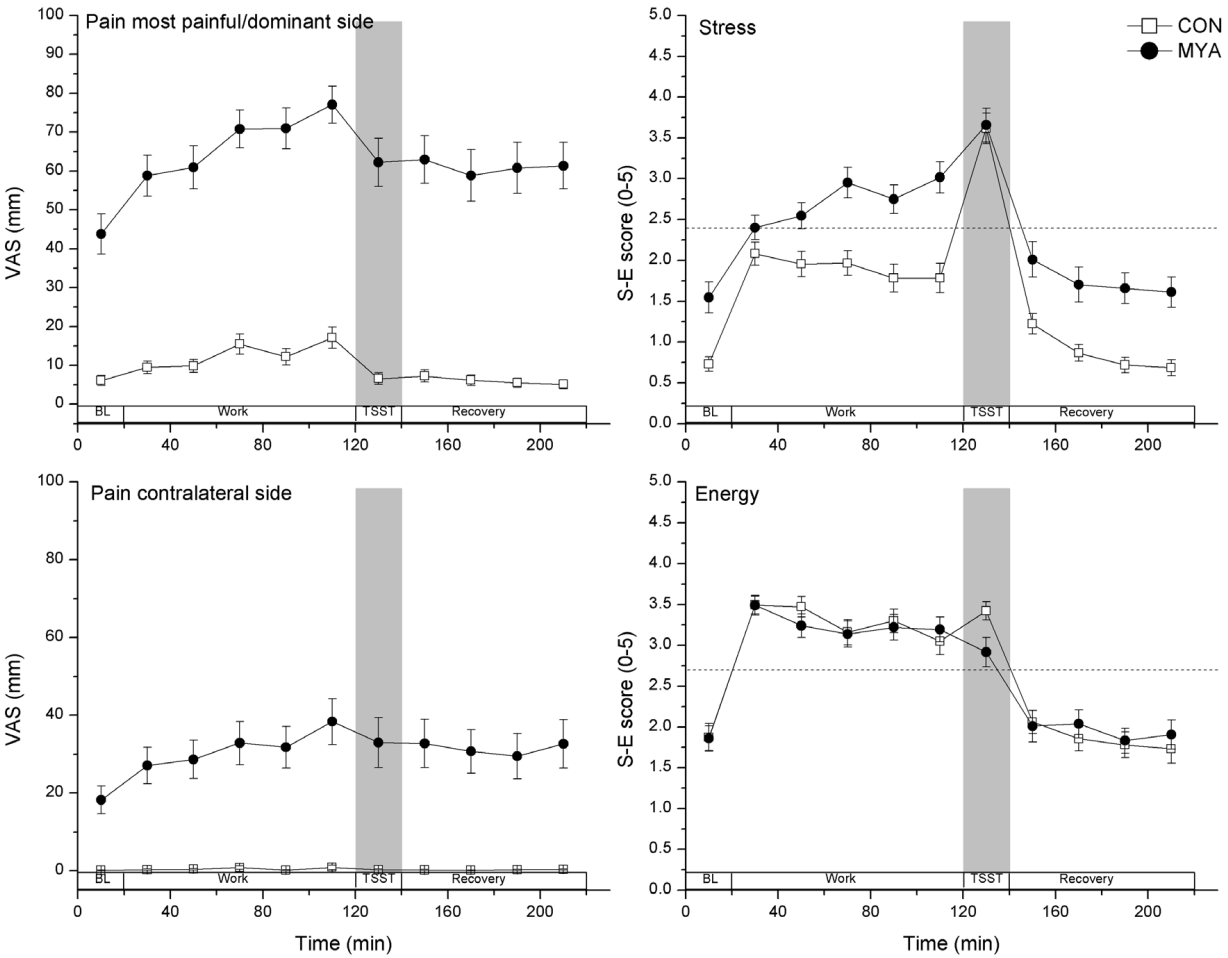
**Subjective ratings (mean ± SEM) during Baseline (BL), work, TSST and recovery periods**. The neutral points for Stress and Energy are indicated by the horizontal dashed lines.

In the mixed model analyses, group (F_1,45 _= 135.8, p < .001), time (F_10,244 _= 22.5, p < .001) and interaction (F_10,244 _= 4.8, p < .001) effects were significant for pain ratings in the dominant/most painful side. The main effect of group was 57.8 mm (95% CI, 47.3 to 68.2 mm) for PAINdomp, i.e., the dominant/most painful side. In the MYA group, PAINdomp continuously increased during the low-force work, peaking at the end of the work period, followed by a slight reduction during TSST and a slow decrease during recovery (Figure 3), never returning to baseline levels. The pain level at the end of the work period was significantly higher than baseline, the first work period, and the three last recovery periods. Looking only at the CON group, none of the time points showed pain ratings significantly higher than baseline level.

For PAINclat, there were significant main effects of group (F_1,44 _= 45.7, p < .001) and time (F_10,212 _= 6.9, p < .001), as well as a significant group × time interaction (F_10,212 _= 5.7, p < .001). The main effect of group was 28.6 mm (95% CI, 19.6 to 37.6). Pain intensity ratings increased significantly from Start work to End work. PAINclat increased significantly from baseline to the third work period, further increased during the remaining work periods and then decreased very slowly towards the end of the experiment in the MYA group. PAINclat was never significantly above zero in the CON group.

For the most painful side, pain ratings increased from 44 mm at Baseline to 77 mm at the end of work and for the contralateral side the corresponding increase was from 18 mm to 38 mm.

#### Stress-Energy ratings

The group (F_1,45 _= 20.3, p < .001), time (F_10,245 _= 74.0, p < .001) and interaction (F_10,245 _= 4.7, p < .001) effects were all statistically significant for Stress ratings. The main effect of group was .84 S-E points (95% CI, .4 to 1.3). The MYA group had higher stress ratings than controls at baseline, work periods 3, 4 and 5, and during recovery. For both groups taken together, stress ratings were significantly higher during the work periods and TSST, compared with Baseline and recovery periods (Figure 3). Stress ratings at TSST were significantly higher than all other time points for both groups. Moreover, stress increased in MYA during the work period, whereas CON became more relaxed towards the end of work. CON were below the neutral point (S-E score 2.4, neither stressed nor relaxed) during the entire work period. MYA, however, started near the neutral point and ended above it. Stress ratings increased to almost identical levels in MYA and CON during the stress test, indicating that the TSST was perceived as equally stressful by both groups. Differences emerged again during recovery where MYA were more stressed, although both groups were below the neutral point and reported stress ratings similar to their respective baseline levels.

Energy ratings did not differ between the two groups (F_1,44 _= .6, p = .434). However, the main effect of time was significant (F_10,267 _= 50.6, p < .001). Energy ratings were significantly higher during work and TSST compared with Baseline and recovery (Figure 3).

#### EMG

The mixed model analyses revealed larger trapezius muscle activity in MYA than in CON (F_1,35 _= 6.2, p = .017) and a significant time effect (F_10,206 _= 18.1, p < .001). The group effect for EMGtrap was 1.7 μV (1.1 to 2.6). The group effect of EMGtrap derived from the mixed model analysis may seem small since the analyses were made using ln-transformed EMG measurements. However, the estimated mean EMGtrap derived from the analysis was 47.4 μV and 27.9 μV for MYA and CON, respectively. For both groups, the muscle activity increased over time during the repetitive work and was significantly elevated compared with baseline, TSST and the last recovery period (Figure 2). The muscle activity then decreased when the subjects performed the TSST. During recovery, EMGtrap first increased to levels similar to the first work period and then decreased over time, ending at levels similar to baseline at the last recovery period.

The deltoid muscle activity was not significantly different between MYA and CON in the mixed model analyses (F_1,67 _= .4, p = .536). The time effect was, however, significant for EMGdelt (F_10,293 _= 70.0, p < .010), showing higher muscle activity during work compared with baseline and TSST. EMGdelt was higher than baseline level at the start of recovery and did not return to baseline level until the last recovery period.

The interaction effect between group and time was not significant for the mixed model analyses involving EMG data and was therefore not included in the models.

#### Autonomic responses

**Figure 4:**
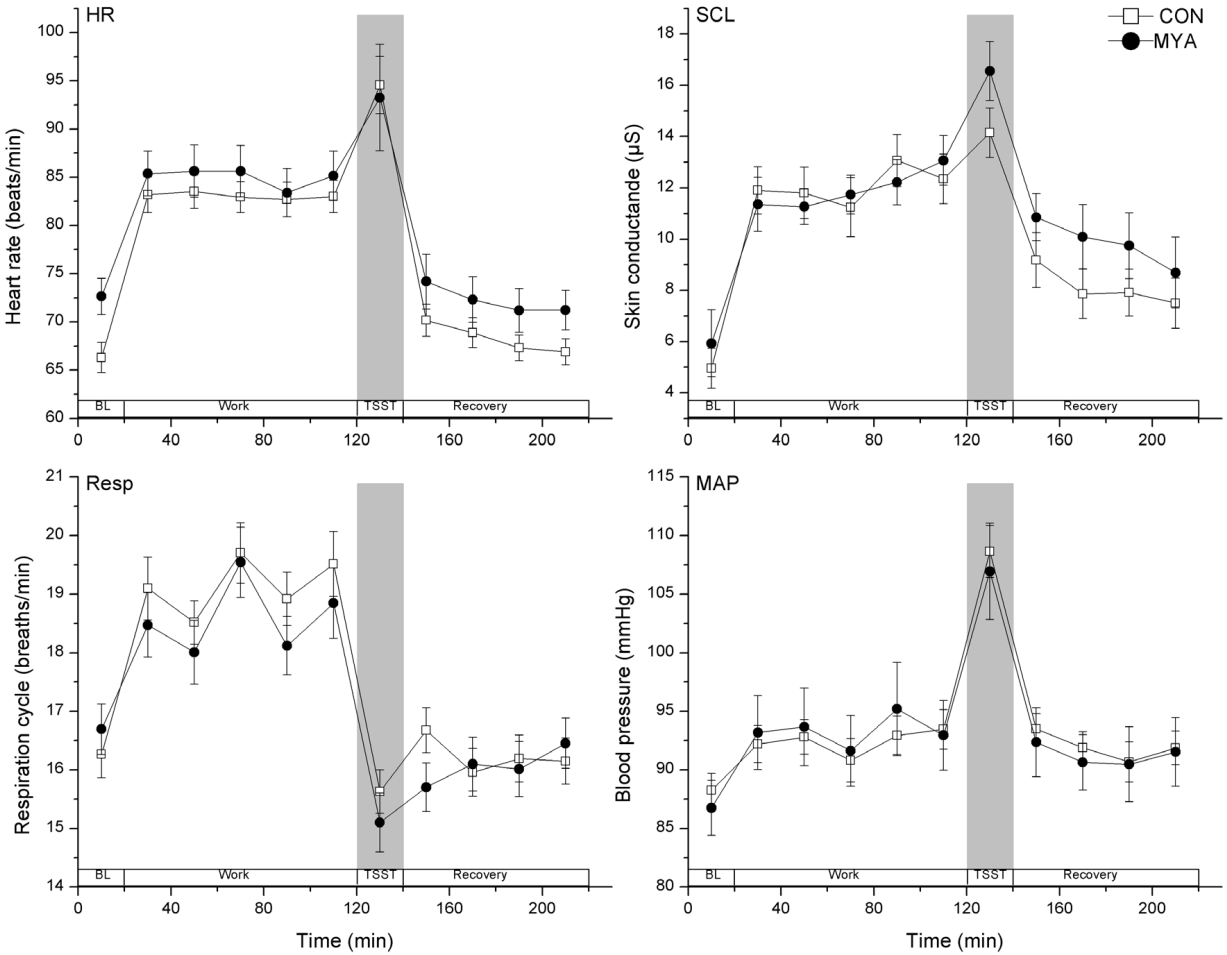
**Physiological measurements (mean ± SEM) during Baseline (BL), work, TSST and recovery periods**.

In the mixed models analysis, HR showed a significant time effect (F_10,387 _= 71.4, p < .001), but group differences were not significant (F_1,73 _= 1.0, p = .332). HR was significantly higher during work and TSST compared with Baseline and recovery (Figure 4). It was also significantly higher during TSST compared with the other time points. HR returned to baseline level within the first 20 min of recovery for both groups.

There was no significant difference in SCL between the groups (F_1,66 _= .04, p = .851). The time effect was significant (F_10,373 _= 27.3, p < .001), with higher skin conductance levels during work and TSST compared with Baseline and the second half of recovery (Figure 4). In addition, SCL was higher during TSST than all other time points. SCL was significantly elevated, compared with baseline, during the first recovery period.

Resp was similar in the two groups, resulting in no significant group effects (F_1,92 _= .1, p = .804). There was, however, a significant time effect for Resp (F_10,343 _= 25.5, p < .001) showing higher respiration rates during work compared with Baseline, TSST and recovery (Figure 4).

The MYA and CON groups showed no significant differences in MAP (F_1,79 _= .2, p = .623). The time effect was significant for MAP (F_10,348 _= 27.0, p < .001). Blood pressure did not rise significantly above baseline or recovery levels during the repetitive work (Figure 4). However, this measurement showed significantly higher values at TSST compared with all other time points.

### Correlations

In MYA, correlations between pain in the dominant/most painful side and the contralateral side were high (rho = .54 to .74, p < .001 to .025) during TSST and recovery. The pain ratings were not significantly correlated during baseline and work. As shown in Table 3, stress and pain ratings were positively correlated during the first three recovery periods and during the third work period. Moreover, EMGtrap was significantly correlated with pain intensity at the first and third recovery (Table 3). In addition, stress and EMGtrap were significantly correlated only at the end of recovery (Table 3). Possible significant correlations between EMGtrap and ratings of pain and stress in the CON group were checked for but not found. Furthermore, no significant correlations were found between autonomic variables and pain intensity or stress in the two groups.

**Table 3:** Correlations between pain intensity, stress ratings and trapezius muscle activity (EMGtrap) for the MYA group.

	**Stress and PAINdomp**	**p-value**	**EMGtrap and PAINdomp**	**p-value**	**EMGtrap and** **Stress**	**p-value**
**Baseline**	0.05	>.3	0.25	>.3	0.45	0.061
**Work 1**	-0.07	>.3	0.32	0.199	-0.21	>.3
**Work 2**	0.26	>.3	0.10	>.3	-0.26	0.288
**Work 3**	0.52	**0.028**	0.14	>.3	-0.13	>.3
**Work 4**	0.16	>.3	0.24	>.3	0.06	>.3
**Work 5**	0.35	0.151	0.28	0.289	-0.37	0.157
**TSST**	0.27	0.275	0.37	0.241	0.48	0.113
**Recovery 1**	0.63	**0.006**	0.54	**0.048**	0.26	>.3
**Recovery 2**	0.57	**0.014**	0.53	0.052	0.38	0.184
**Recovery 3**	0.54	**0.020**	0.54	**0.037**	0.41	0.126
**Recovery 4**	0.10	>.3	0.18	>.3	0.62	**0.019**

## Discussion

The main finding in the present study was that muscle activity in the painful muscle was higher in a group of women with trapezius myalgia compared with pain-free controls. Furthermore, muscle activity in MYA was positively correlated to pain intensity during recovery. The significant increases in HR, MAP and SCL during the stress test and the subsequent reduction during recovery demonstrates that stress was successfully induced. Unexpectedly, the two groups showed very similar autonomic responses to low-force repetitive work and psychosocial stress. This finding does not support the hypothesis that chronic trapezius myalgia is associated with increased sympathetic activity. The perceived level of stress was, however, increased in the patient group during resting and work conditions, compared with controls.

### Pain and stress ratings

As expected and intended, the trapezius myalgia group reported more pain than controls throughout the experiment. Pain intensity ratings confirmed that the low-force work had the desired effect, i.e., it worsened the pain considerably for MYA, whereas pain ratings for the CON group never rose significantly above baseline level.

Our results regarding pain intensity and stress ratings during the psychosocial stress test are inconsistent with the findings by Thieme et al. [38] that stress enhanced pain intensity in fibromyalgia syndrome (FMS). The MYA group's reduction in pain intensity at the end of the psychosocial stress test and subsequent increase at the first recovery period could not be explained only by cessation of the repetitive work tasks. This reduction in pain intensity may be due to stress-induced analgesia [39] and/or distraction from the sensation of pain [40].

MYA experienced higher level of stress at baseline compared with CON, which is consistent with previous findings in chronic pain patients by Thieme et al. [38]. Chapman and Gavrin [41] concluded that pain is a powerful stressor per se, which would implicate that during the experiment, MYA were exposed to two different stressors simultaneously; pain and psychosocial stress. The increasing stress ratings in MYA during the work period could, thus, be due to increased pain. There are indications of attenuated responses to stress and functional tests in FMS [42]. A possible attenuation of the stress response to the TSST could, hence, be masked in the additional stress due to pain in the MYA group. However, introducing pain intensity as a covariate in the analyses, thus controlling for differences in pain ratings, did not support such a suggestion.

In contrast to CON, the MYA group's energy level actually decreased in response to the stress test which could indicate that the chronic pain group perceives stressful situations as being more energy consuming. The CON group found ways to mobilize energy for the stress test, which was seen as increased energy ratings at TSST.

### Muscle activity

Similar patterns of EMG activity in the prime mover deltoid were found in MYA and CON throughout the experiment (Figure 2). This was not the case for the postural trapezius muscle.

The chronic pain group's muscle activity was higher in the painful trapezius muscle at baseline, which was an uninstructed rest, but not at the reference relaxation measurement where the subjects were given specific instructions on how to relax the trapezius muscle. Speculatively, the higher trapezius EMG in MYA during uninstructed rest, but not when instructed to fully relax, can be an indication that voluntary effort and attention are required in order to relax the muscle. In daily regular activities, when other aspects need attention, the EMG activity will be higher, contributing to a vicious circle of the muscle. Our results need to be confirmed in other studies but opens up for myofeedback interventions using surface EMG. Hitherto such myofeedback studies indicate that it is possible to alter EMG patterns in healthy subjects [43] and in chronic myalgia [44]. Although the latter study did not find that the EMG level per se – but rather the ability to relax – was linked to pain intensity decrease [44].

In agreement with other studies we found that the MYA group's muscle activity of the trapezius was higher throughout the experiment [45-47]; there exist different explanations for the higher muscle activity in subjects with chronic pain [48,49]. The direction of the causal relationship between pain and EMG activity is not obvious [49] and in fact cannot be determined using the present study design. We do not know if the increased muscle activity is a cause or a consequence of pain, since it is unknown whether this was also the case before pain onset. The literature is, however, not in consensus and some studies have found no differences in EMG activity [50-52]. In contrast, we found no difference in the EMG activity of the prime mover, i.e., the deltoid, between the two groups of subjects. There are reports that subjects with pain have difficulties in relaxing their trapezius after completion of a task [53], in utilizing small pauses in work [54,55] and in parts of the contraction cycle during dynamic muscle contractions [10-16], but the literature is not in consensus [50,56-58]. With the present study design, it was not possible to determine whether the EMG activity of trapezius in MYA was increased during the active contraction, during the supposed passive parts of the contraction cycles, or during both these.

The increase in EMG activity of the trapezius in response to work was similar in MYA and CON. A successive increase in EMG activity of trapezius during work has also been reported by Strøm et al. [59]. Furthermore, muscle activity was not correlated to pain development during repetitive work. Hence, muscle activity per se does not seem to be the cause for increased pain intensity. Strøm et al. [59] reported similar results but also that the increase in pain intensity correlated with muscle blood flux. It cannot be ruled out that higher EMG activity in trapezius can affect blood flow and microcirculation in MYA.

### Autonomic responses

None of the autonomic responses showed any signs of an increased sympathetic activity in MYA. Reactions to the low-force work and TSST were almost identical in the two groups.

The higher baseline HR found in MYA confirms previous findings by Gockel et al. [60]. Regarding baseline differences, a previous studie on patients with chronic low back pain (CLBP) found higher HR, higher low frequency heart rate variability (HRV) and lower high frequency HRV [61]. In contrast to the study referred to above, there are several studies that fail to detect differences between CLBP patients and controls in baseline levels [62,63]. Flor and Turk [64] concluded that regardless of type of physiological measure, baseline levels were not generally elevated in CLBP patients. Patients with whiplash associated disorders (WAD) have shown increased heart rate and decreased heart rate variability (HRV) [22] at baseline. The basal autonomic state in widespread pain has been more extensively investigated. Cohen et al. [25] found increased sympathetic and decreased parasympathetic activity during complete rest in the supine position. The results are, however, conflicting even for FMS patients but some sort of dysautonomia has been found in most studies [38,42]. The elevated baseline HR found in this study should probably not be interpreted as a sign of increased sympathetic tone since none of the other measures gave similar indications and HR can be influenced by other factors e.g., physical fitness.

Our findings of normal cardiovascular reactivity in MYA are in contrast to the study by Gockel et al. [60], where patients with neck/shoulder pain showed attenuated cardiovascular responses to various functional tests (paced breathing, orthostatic, handgrip, valsalva). The results from earlier studies are inconsistent reporting both increased and decreased cardiovascular reactivity in chronic pain patients compared with controls. In a study comparing WAD and controls [22], unpleasant sound induced higher HR response in WAD, HR and HRV reactivity was lower in response to cognitive challenge (Stroop color-word test) whereas no differences emerged during paced breathing. No difference in autonomic reactivity to various tests (Stroop color-word, orthostatic, handgrip, paced breathing) was found between CLBP and controls [61]. Nilsen et al. [48] studied differences between neck/shoulder pain patients, FMS patients and healthy controls. They found attenuated cardiovascular reactions to a low-grade mental stressor in FMS. Neck/shoulder pain patients appeared to be in an intermediate position between FMS and controls, but not significantly different from the controls. FMS patients have also shown normal blood pressure and HR regulation during maximal voluntary contractions [65] and lower HR reactivity to stressors (mental arithmetic, social conflict) [38].

Skin conductance data in the present study showed tendencies towards an increased stress response and delayed return to baseline in MYA, but the difference between groups was small and individual variations large. Higher SCL in response to stress and during recovery from stress, which indicates enhanced sympathetic response to stress, has previously been found in FMS [38]. Higher spontaneous electrodermal activity [61] and enhanced activity in response to pain [62] and sound stimulation [66] has also been found in CLBP patients.

The chronic pain group in this study did not have widespread pain but local or regional pain in the neck/shoulder area. The autonomic dysfunctions found in FMS could be due to the alterations at different levels of the pain system in this generalized pain syndrome [67]. Alterations in cardiovascular or sudomotor regulations could be present in local or regional pain as well, but in this case localized only to the painful areas, as seen in complex regional pain syndromes [68].

Inconsistencies in the results from previous studies of autonomic reactivity in patients with localized chronic pain may be due to the heterogeneity of this patient group. They represent a diversity of causes, localizations and intensities of pain. The findings in FMS, WAD and CLBP discussed above may reflect nonspecific response patterns characteristic of confounding co-morbidities, e.g., anxiety or depression, in general, which was not found in the present MYA population.

### Methodological considerations

Previous studies concerning stress responses in chronic pain have used cognitive tasks (e.g., Stroop color-word test, mental arithmetic) or social conflict tasks, which are not as powerful as the TSST in producing acute stress responses [69]. This stress protocol has been found to induce significant cardiovascular responses at the first exposure in 70–80% of all subjects [70]. Laboratory stress protocols offer the advantage of standardization across test sessions but may lack the ecological validity of field studies. However, the significant increases in HR, MAP, SCL and subjective stress ratings confirm that psychosocial stress was successfully induced.

The autonomic measures used here, i.e., instantaneous heart rate, skin conductance, and blood pressure only provide gross estimates of end organ function, irrespective of the relative contribution of the sympathetic and parasympathetic branches. Simple examination of autonomic responses to challenges does not provide a definitive test of the potential role of the sympathetic nervous system. More sophisticated methods, such as heart rate variability (HRV), could provide information regarding the balance of the autonomic branches. However, in the present study, the subjects assumed different body positions, i.e., both sitting and standing, and were moving, circumstances that affects HRV and makes it difficult to interpret. Moreover, valuable information about interactions between different variables could be derived through multivariate data analyses, possibly revealing response patterns not visible when analyzing one variable at a time.

Inclusion of only female subjects was based on the higher prevalence of chronic trapezius myalgia in women than men, also limiting the availability of eligible male subjects. Previous studies have concluded that healthy men and women are similar in their autonomic responses to psychosocial stress tests [71,72]. There are, however, sex differences in chronic pain (e. g., CLBP) patients' reactions to experimental pain [73]. Including men is therefore important for future research.

A limitation of the present study is that the statistical power did not allow differences below approximately 10% in any of the outcome variables to be detected, increasing the risk for statistical type II error. Correction of p-values for multiple comparisons, to avoid type I error, is often performed in situations with multiple outcome variables. However, because of the explorative approach in this study, correction for multiple comparisons were only performed in the mixed model post-hoc analyses, to reduce the risk of type II error in the between groups tests [74]. The pain intensity distributions were somewhat skewed, implying that the results from the mixed regression model should be interpreted with care. However, residuals from the analyses were approximately symmetrically distributed, the differences between groups were large and log- or square root-transformation of the variables did not alter the results indicating that the conclusions regarding pain ratings were valid.

## Conclusion

We found increased muscle activity during uninstructed rest in the painful muscle of a group of women with trapezius myalgia. The present study could not confirm the hypothesis that chronic trapezius myalgia is associated with increased sympathetic activity. The suggestion of autonomic imbalance in patients with chronic local or regional musculoskeletal pain needs to be further investigated.

## Competing interests

The authors declare that they have no competing interests.

## Authors' contributions

AS carried out the data collection, performed the statistical analysis and was the main writer of the manuscript. BL designed the study, participated in subject inclusion and data collection and helped to draft the manuscript. JD participated in the planning and coordination of the study, and revised the manuscript. TF was involved in methodological considerations and revised the manuscript. BG designed and supervised the study and helped to draft the manuscript. All authors read and approved the final manuscript.

## Pre-publication history

The pre-publication history for this paper can be accessed here:



## References

[B1] Flor H, Birbaumer N (1994). Acquisition of chronic pain: Psychophysiological mechanisms. Am Pain Soc J.

[B2] Passatore M, Roatta S (2006). Influence of sympathetic nervous system on sensorimotor function: whiplash associated disorders (WAD) as a model. Eur J Appl Physiol.

[B3] Ariëns GA, van Mechelen W, Bongers PM, Bouter LM, Wal G van der (2001). Psychosocial risk factors for neck pain: A systematic review. Am J Ind Med.

[B4] Malchaire J, Cock N, Vergracht S (2001). Review of the factors associated with musculoskeletal problems in epidemiological studies. Int Arch Occup Environ Health.

[B5] Bongers PM, Kremer AM, Laak Jt (2002). Are psychosocial factors, risk factors for symptoms and signs of the shoulder, elbow, or hand/wrist?: A review of the epidemiological literature. Am J Ind Med.

[B6] Wærsted M (2000). Human muscle activity related to non-biomechanical factors in the workplace. Eur J Appl Physiol.

[B7] Gerdle B, Karlsson S, Day S, Djupsjöbacka M, Johansson H, Winhorst U (1999). Acquisition, processing and analysis of surface EMG signals. Modern Techniques in Neuroscience Research.

[B8] Madeleine P, Leclerc F, Arendt-Nielsen L, Ravier P, Farina D (2006). Experimental muscle pain changes the spatial distribution of upper trapezius muscle activity during sustained contraction. Clin Neurophysiol.

[B9] Falla D, Farina D, Graven-Nielsen T (2007). Experimental muscle pain results in reorganization of coordination among trapezius muscle subdivisions during repetitive shoulder flexion. Exp Brain Res.

[B10] Elert J, Kendall SA, Larsson B, Mansson B, Gerdle B (2001). Chronic pain and difficulty in relaxing postural muscles in patients with fibromyalgia and chronic whiplash associated disorders. J Rheumatol.

[B11] Elert JE, Rantapaa Dahlqvist SB, Henriksson-Larsen K, Gerdle B (1989). Increased EMG activity during short pauses in patients with primary fibromyalgia. Scand J Rheumatol.

[B12] Elert JE, Rantapaa-Dahlqvist SB, Henriksson-Larsen K, Lorentzon R, Gerdle BU (1992). Muscle performance, electromyography and fibre type composition in fibromyalgia and work-related myalgia. Scand J Rheumatol.

[B13] Arendt-Nielsen L, Graven-Nielsen T, Svarrer H, Svensson P (1996). The influence of low back pain on muscle activity and coordination during gait: a clinical and experimental study. Pain.

[B14] Fredin Y, Elert J, Britschgi N, Vaher A, Gerdle B (1997). A decreased ability to relax between repetitive muscle contractions in patients with chronic symptoms after whiplash trauma of the neck. J Musculoskel Pain.

[B15] Graven-Nielsen T, Svensson P, Arendt-Nielsen L (1997). Effects of experimental muscle pain on muscle activity and co-ordination during static and dynamic motor function. Electroen Clin Neurophysiol.

[B16] Stohler CS, Ashton-Miller JA, Carlson DS (1988). The effects of pain from the mandibular joint and muscles on masticatory motor behaviour in man. Arch Oral Biol.

[B17] Lund JP, Donga R, Widmar CG, Stohler CS (1990). The pain-adaptation model: a discussion of the relationship between chronic musculoskeletal pain and motor activity. Can J Physiol Pharmacol.

[B18] Andersen LL, Nielsen PK, Søgaard K, Andersen CH, Skotte J, Sjøgaard G (2008). Torque-EMG-velocity relationship in female workers with chronic neck muscle pain. J Biomech.

[B19] Lundberg U, Forsman M, Zachau G, Eklöf M, Palmerud G, Melin B, Kadefors R (2002). Effects of experimentally induced mental and physical stress on motor unit recruitment in the trapezius muscle. Work Stress.

[B20] Lundberg U, Dohns IE, Melin B, Sandsjö L, Palmerud G, Kadefors R, Ekström M, Parr D (1999). Psychophysiological Stress Responses, Muscle Tension, and Neck and Shoulder Pain Among Supermarket Cashiers. J Occup Health Psych.

[B21] Johansson H, Arendt-Nilsen L, Bergenheim M, Blair S, van Dieen JH, Djupsjöbacka M, Fallentin N, Gold JE, Hägg G, Kalezic N, Larsson S-E, Ljubisavljevic M, Lyskov E, Mano T, Magnusson M, Passatore M, Pedrosa-Domellöf F, Punnett L, Roatta S, Thornell L-E, Windhorst U, Zukowska Z, Johansson H, Windhorst U, Djupsjöbacka M, Passatore M (2003). Epilogue: An Integrated Model for Chronic Work-Realted Myalgia – "Brussels Model". Chronic Work-Related Myalgia – Neuromuscular Mechanisms behind Work-Related Chronic Muscle Pain Syndromes.

[B22] Kalezic N (2006). Autonomic Reactivity in Muscle Pain – Clinical and Experimental Assessment. Doctoral thesis.

[B23] Roatta S, Arendt-Nielsen L, Farina D (2008). Sympathetic-induced changes in discharge rate and spike-triggered average twitch torque of low-threshold motor units in humans. J Physiol.

[B24] Roatta S, Kalezic N, Passatore M, Johansson H, Windhorst U, Djupsjöbacka M, Passatore M (2003). Sympathetic Nervous System: Interaction with Muscle Function and Involvement in Motor Control. Chronic work-related myalgia.

[B25] Cohen H, Neumann L, Shore M, Amir M, Cassuto Y, Buskila D (2000). Autonomic dysfunction in patients with fibromyalgia: Application of power spectral analysis of heart rate variability. Semin Arthritis Rheum.

[B26] Martinez-Lavin M (2007). Biology and therapy of fibromyalgia. Stress, the stress response system, and fibromyalgia. Arthritis Res Ther.

[B27] Gockel M, Lindholm H, Niemistö L, Hurri H (2008). Perceived disability but not pain is connected with autonomic nervous function among patients with chronic low back pain. J Rehabil Med.

[B28] Ohlsson K, Attewell RG, Johnsson B, Ahlm A, Skerfving S (1994). An assessment of neck and upper extremity disorders by questionnaire and clinical examination. Ergonomics.

[B29] Kuorinka I, Jonsson B, Kilbom Å, Vinterberg H, Biering-Sørensen F, Andersson G, Jørgensen K (1987). Standardised Nordic questionnaires for the analysis of musculoskeletal symptoms. Appl Ergon.

[B30] Wolfe F, Smythe HA, Yunus MB, Bennett RM, Bombardier C, Goldenberg DL, Tugwell P, Campbell SM, Abeles M, Clark P, Fam AG, Farber SJ, Fiechtner JJ, Franklin CM, Gatter RA, Hamaty D, Lessard J, Lichtbroun AS, Masi AT, Mccain GA, Reynolds WJ, Romano TJ, Russell IJ, Sheon RP (1990). The american college of rheumatology 1990 criteria for the classification of fibromyalgia. Arthritis Rheum.

[B31] Larsson B, Rosendal L, Kristiansen J, Sjøgaard G, Søgaard K, Ghafouri B, Abdiu A, Kjaer M, Gerdle B (2008). Responses of algesic and metabolic substances to 8 h of repetitive manual work in myalgic human trapezius muscle. Pain.

[B32] Rosendal L, Larsson B, Kristiansen J, Peolsson M, Sogaard K, Kjaer M, Sorensen J, Gerdle B (2004). Increase in muscle nociceptive substances and anaerobic metabolism in patients with trapezius myalgia: microdialysis in rest and during exercise. Pain.

[B33] Kirschbaum C, Pirke KM, Hellhammer DH (1993). The 'Trier Social Stress Test' – a tool for investigating psychobiological stress responses in a laboratory setting. Neuropsychobiology.

[B34] Kirschbaum C, Kudielka BM, Gaab J, Schommer NC, Hellhammer DH (1999). Impact of Gender, Menstrual Cycle Phase, and Oral Contraceptives on the Activity of the Hypothalamus-Pituitary-Adrenal Axis. Psychosom Med.

[B35] Kjellberg A, Wadman C (2007). The role of the affective stress response as a mediator of the effect of psychosocial risk factors on musculoskeletal complaints – Part 1: Assembly workers. Int J Ind Ergon.

[B36] Kjellberg A, Wadman C (2002). Subjektiv stress och dess samband med psykosociala arbetsförhållanden och hälsobesvär. En prövning av Stress-Energi-modellen. [Subjective stress and its relation to psychosocial work conditions and health complaints. A test of the Stress-Energy model]. Arbete och Hälsa.

[B37] Hermens HJ, Fredriks B, Merletti R, Stegeman D, Blok J, Rau G, Disselhorst-Klug C, Hägg G (1999). SENIAM European recommendations for surface elecromyography.

[B38] Thieme K, Rose U, Pinkpank T, Spies C, Turk DC, Flor H (2006). Psychophysiological responses in patients with fibromyalgia syndrome. J Psychosom Res.

[B39] Amit Z, Galina ZH (1986). Stress-induced analgesia: adaptive pain suppression. Physiol Rev.

[B40] Quiton RL, Greenspan JD (2007). Sex differences in endogenous pain modulation by distracting and painful conditioning stimulation. Pain.

[B41] Chapman CR, Gavrin J (1999). Suffering: the contributions of persistent pain. Lancet.

[B42] Cohen H, Neumann L, Kotler M, Buskila D (2001). Autonomic nervous system derangement in fibromyalgia syndrome and related disorders. IMAJ.

[B43] Holtermann A, Søgaard K, Christensen H, Dahl B, Blangsted A (2008). The influence of biofeedback training on trapezius activity and rest during occupational computer work: a randomized controlled trial. Eur J Appl Physiol.

[B44] Vollenbroek-Hutten M, Hermens H, Voerman G, Sandsjö L, Kadefors R (2006). Are changes in pain induced by myofeedback training related to changes in muscle activation patterns in patients with work-related myalgia?. Eur J Appl Physiol.

[B45] Johnston V, Jull G, Souvlis T, Jimmieson NL (2008). Neck Movement and Muscle Activity Characteristics in Female Office Workers With Neck Pain. Spine.

[B46] Szeto GPY, Straker LM, O'Sullivan PB (2005). A comparison of symptomatic and asymptomatic office workers performing monotonous keyboard work – 1: Neck and shoulder muscle recruitment patterns. Manual Therapy.

[B47] Falla D, Bilenkij G, Jull G (2004). Patients with chronic neck pain demonstrate altered patterns of muscle activation during performance of a functional upper limb task. Spine.

[B48] Nilsen KB, Sand T, Westgaard RH, Stovner LJ, White LR, Bang Leistad R, Helde G, Rø M (2007). Autonomic activation and pain in response to low-grade mental stress in fibromyalgia and shoulder/neck pain patients. Eur J Pain.

[B49] Larsson B, Björk J, Elert J, Gerdle B (2000). Mechanical performance and electromyography during repeated maximal isokinetic shoulder forward flexions in female cleaners with and without myalgia of the trapezius muscle and in healthy controls. Eur J Appl Physiol.

[B50] Voerman G, Vollenbroek-Hutten M, Hermens H (2007). Upper trapezius muscle activation patterns in neck-shoulder pain patients and healthy controls. Eur J Appl Physiol.

[B51] Westgaard RH, Vasseljen O, Holte KA (2001). Trapezius muscle activity as a risk indicator for shoulder and neck pain in female service workers with low biomechanical exposure. Ergonomics.

[B52] Larsson S-E, Ålund M, Cai H, Öberg ÅP (1994). Chronic pain after soft-tissue injury of the cervical spine: trapezius muscle blood flow and electromyography at static loads and fatigue. Pain.

[B53] Johnston V, Jull G, Darnell R, Jimmieson N, Souvlis T (2008). Alterations in cervical muscle activity in functional and stressful tasks in female office workers with neck pain. Eur J Appl Physiol.

[B54] Hägg G, Åström A (1997). Load pattern and pressure pain threshold in the upper trapezius muscle and psychosocial factors in medical secretaries with and without shoulder/neck disorders. Int Arch Occup Environ Health.

[B55] Sandsjö L, Melin B, Rissén D, Dohns I, Lundberg U (2000). Trapezius muscle activity, neck and shoulder pain, and subjective experiences during monotonous work in women. Eur J Appl Physiol.

[B56] Nordander C, Hansson G-A (2000). Muscular rest and gap frequency as EMG measures of physical exposure: the impact of work tasks and individual related. Ergonomics.

[B57] Vasseljen O, Westgaard RH (1995). A case-control study of trapezius muscle activity in office and manual workers with shoulder and neck pain and symptom-free controls. Int Arch Occup Environ Health.

[B58] Thorn S, Søgaard K, Kallenberg LAC, Sandsjö L, Sjøgaard G, Hermens HJ, Kadefors R, Forsman M (2007). Trapezius muscle rest time during standardised computer work – A comparison of female computer users with and without self-reported neck/shoulder complaints. J Electromyogr Kines.

[B59] Strøm V, Knardahl S, Stanghelle JK, Røe C Pain induced by a single simulated office-work session: Time course and association with muscle blood flux and muscle activity. Eur J Pain.

[B60] Gockel M, Lindholm H, Alaranta H, Viljanen A, Lindquist A, Lindholm T (1995). Cardiovascular functional disorder and stress among patients having neck-shoulder symptoms. Ann Rheum Dis.

[B61] Kalezic N, Åsell M, Kerschbaumer H, Lyskov E (2007). Physiological reactivity to functional tests in patients with chronic low back pain. J Musculoskelet Pain.

[B62] Peters ML, Schmidt AJM (1991). Psychophysiological responses to repeated acute pain stimulation in chronic low back pain patients. J Psychosom Res.

[B63] Cohen MJ, Swanson GA, Naliboff BD, Schandler SL, McArthur DL (1986). Comparison of electromyographic response patterns during posture and stress tasks in chronic low back pain patterns and control. J Psychosom Res.

[B64] Flor H, Turk DC (1989). Psychophysiology of Chronic Pain: Do Chronic Pain Patients Exhibit Symptom-Specific Psychophysiological Responses?. Psychol Bull.

[B65] Kadetoff D, Kosek E (2007). The effects of static muscular contraction on blood pressure, heart rate, pain ratings and pressure pain thresholds in healthy individuals and patients with fibromyalgia. Eur J Pain.

[B66] Bonnet A, Naveteur J (2004). Electrodermal activity in low back pain patients with and without co-morbid depression. Int J Psychophysiol.

[B67] Vierck CJJ (2006). Mechanisms underlying development of spatially distributed chronic pain (fibromyalgia). Pain.

[B68] Baron R, Levine JD, Fields HL (1999). Causalgia and reflex sympathetic dystrophy: Does the sympathetic nervous system contribute to the generation of pain?. Muscle Nerve.

[B69] Dickerson SS, Kemeny ME (2004). Acute stressors and cortisol reactivity: A theoretical integration and synthesis of laboratory research. Psychol Bull.

[B70] Schommer NC, Hellhammer DH, Kirschbaum C (2004). Dissociation between reactivity of the hypothalamus-pituitary-adrenal axis and the sympathetic-adrenal-medullary system to repeated psychosocial stress. Psychosom Med.

[B71] Kelly MM, Tyrka AR, Anderson GM, Price LH, Carpenter LL (2008). Sex differences in emotional and physiological responses to the Trier Social Stress Test. J Behav Ther Exp Psychiatry.

[B72] Sgoifo A, Braglia F, Costoli T, Musso E, Meerlo P, Ceresini G, Troisi A (2003). Cardiac autonomic reactivity and salivary cortisol in men and women exposed to social stressors: relationship with individual ethological profile. Neurosci Biobehav Rev.

[B73] Tousignant-Laflamme Y, Marchand S (2006). Sex differences in cardiac and autonomic response to clinical and experimental pain in LBP patients. Eur J Pain.

[B74] Feise R (2002). Do multiple outcome measures require p-value adjustment?. BMC Med Res Method.

